# Protective value of ischemia-free liver transplantation on post-transplant acute kidney injury

**DOI:** 10.1016/j.jhepr.2025.101339

**Published:** 2025-01-29

**Authors:** Qiang Zhao, Jinbo Huang, Meiting Qin, Yunhua Tang, Zhiying Liu, Yefu Li, Zhiyong Guo, Jia Dan, Yu Nie, Xiaoshun He

**Affiliations:** 1 Organ Transplant Center, The First Affiliated Hospital, Sun Yat-sen University, Guangzhou, China; 2 Guangdong Provincial Key Laboratory of Organ Donation and Transplant Immunology, Guangzhou, China; 3 Guangdong Provincial International Cooperation Base of Science and Technology, Guangzhou, China; 4 Zhongshan School of Medicine, Sun Yat-sen University, Guangzhou, China; 5 General Surgery Center, Department of Hepatobiliary Surgery II, Research Center for Artificial Organ and Tissue Engineering, Guangzhou Clinical Research and Transformation Center for Artificial Liver, Institute of Regenerative Medicine, Zhujiang Hospital, Southern Medical University, Guangzhou, China

**Keywords:** Ischemia-free liver transplantation, Acute kidney injury, Extended criteria donor, Propensity score matching

## Abstract

**Background & Aims:**

Ischemia-free liver transplantation (IFLT) completely avoids ischemia–reperfusion injury (IRI), thus potentially reducing acute kidney injury (AKI) after liver transplantation (LT). Therefore, this study investigated whether IFLT has a protective effect against AKI after LT.

**Methods:**

In total, 862 patients who had undergone LT between 2017 to 2022 were divided into an ischemia-free liver transplantation group (IFLT group) and conventional liver transplantation group (CLT group) based on the surgical methods used. Propensity score matching (PSM) was used for post hoc randomization in the 1:1 matching between the groups. Post-transplant kidney function, graft function, and patient survival were compared between the groups. Multivariate logistic regression analysis was used to identify the risk factors of AKI after LT.

**Results:**

Overall, 745 out of 862 patients were finally enrolled, of whom 98 underwent IFLT. PSM created 94 pairs of patients. IFLT resulted in a significant reduction in Stage-3 AKI (3.2% *vs.* 16.0%, *p* = 0.003), severe AKI (SAKI) (13.8% *vs.* 25.5%, *p* = 0.044), and renal replacement therapy (RRT) ratio (3.2% *vs.* 12.8%, *p* = 0.015) compared with the CLT group. The early allograft dysfunction (EAD) incidence of the IFLT group significantly decreased (8.5% *vs.* 44.7%, *p* <0.001). Livers from the extended criteria donation (ECD) were received in 49 patients who underwent IFLT and 46 patients who underwent CLT. Compared with the ECD-CLT group, the Stage-3 AKI and SAKI incidence in the ECD-IFLT group were both decreased (*p <*0.05). Multivariate logistic regression analysis further confirmed that both using IFLT and avoiding ECD were protective factors for post-transplant Stage-3 AKI.

**Conclusions:**

IFLT significantly reduces the incidence of post-transplant SCKI, Stage-3 AKI, and RRT. Importantly, this protective effect is also present in patients receiving ECD livers.

**Impact and implications:**

Ischemia-free liver transplantation significantly reduces the incidence of severe acute kidney injury, Stage-3 acute kidney injury and renal replacement therapy after liver transplantation. Importantly, this protective effect is also present in patients receiving extended criteria donation livers.

**Clinical trial number:**

ChiCTR2400081755.

## Highlights

• Acute kidney injury is a common complication after liver transplantation
• Ischemia-reperfusion injury is a common cause of post-transplant acute kidney injury
• Ischemia-free liver transplantation has a renoprotective effect.
• The renoprotective effect is also present in extended criteria donor transplantation.

## Graphical abstract

**Figure:**
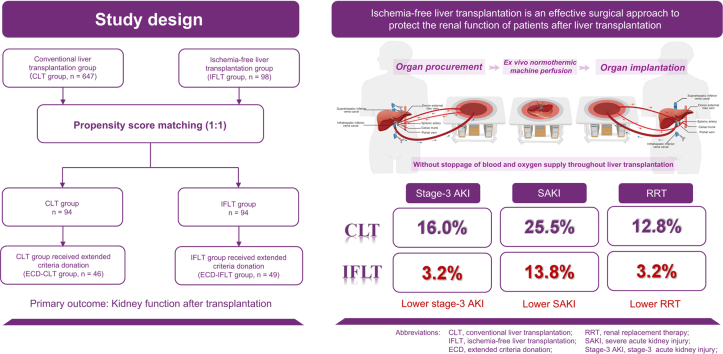

## Introduction

Liver transplantation (LT) has become the preferred treatment for end-stage liver disease.^1^ Acute kidney injury (AKI) is a common complication after LT. Early post-transplant AKI is significantly associated with both short- and long-term adverse outcomes.[2], [3], [4], [5], [6] Furthermore, an increasing number of extended criteria donor (ECD) livers are being used in LT.^7^ ECD organs exhibit lower tolerance to ischemia-reperfusion injury (IRI), leading to a significant increase in the incidence of post-transplant AKI.^8^^,^^9^

Post-transplant AKI is the result of the combined effects of recipient, graft, operation, and postoperative management.^2^^,^^4^ Inevitable during conventional liver transplantation (CLT) procedures, IRI is one of the common causes.[10], [11], [9] The mechanism can involve the systemic inflammatory response syndrome (SIRS) induced by IRI, which damages the renal tubular epithelial and vascular endothelial cells, as well as causing hemodynamic fluctuations and a state of reduced renal perfusion.^10^^,^^12^^,^^13^

For decades, static cold storage (SCS) has been the gold standard for organ preservation during CLT. However, IRI is unavoidable and organ quality suffers significant damage, posing a common technical challenge in the field of organ transplantation. In 2017, our team successfully performed the world's first ‘ischemia-free liver transplantation’ (IFLT).^14^ IFLT is a novel LT technique that establishes continuous normothermic machine perfusion (NMP) by establishing the trifurcation structure of the portal vein, hepatic artery, and inferior vena cava behind the liver.^14^ Throughout the entire process of organ procurement, preservation, and implantation, it provides the liver with blood at physiological temperature, thereby completely avoiding IRI.^14^ Using comprehensive evaluations, such as pathology, transcriptomics, proteomics, metabolomics, single-cell sequencing, and immunology, our team further confirmed that IFLT can avoid characteristic pathological and physiological changes associated with IRI.^15^ Graft IRI not only causes localized graft damage, but also contributes to remote organ injuries affecting the kidneys, heart, lungs, and intestines, thereby triggering systemic inflammatory responses.^16^ By mitigating the systemic inflammation caused by IRI, IFLT helps avoid hemodynamic instability.

In addition to mitigating IRI, IFLT also incorporates the numerous advantages of normothermic machine perfusion (NMP) technology. The flushing effect of machine perfusion during IFLT significantly reduces the inflammatory factors residing in the liver, while IFLT itself greatly reduces the release of inflammatory factors from the liver into the systemic circulation after the liver is implanted.^15^^,^^17^ Moreover, the electrolyte balance is carefully maintained throughout the machine perfusion process. Once the liver is implanted, there is no release of high potassium or other abnormal electrolytes into the systemic circulation that could cause severe electrolyte fluctuations. In addition, IFLT provides the liver with blood at physiological temperature. Recipients who received IFLT did not experience low body temperature, while CLT recipients did. Thus, hemodynamic instability was avoided because IFLT avoids adverse factors causing circulatory fluctuations during transplantation, including inflammatory cytokines, hepatic release of abnormal electrolytes and low body temperature.

IFLT overcomes the two major surgical-related risk factors that contribute to the development of post-transplant AKI: the inflammatory response induced by IRI and renal hypoperfusion caused by hemodynamic instability.^15^^,^^18^^,^^19^ In theory, it could also offer protection against post-transplant AKI. Therefore, the current study systematically explored the protective effects of IFLT on post-LT AKI using a post hoc randomization method, propensity score matching (PSM).

## Method

### Patients and study design

The study retrospectively reviewed patients who underwent LT at the First Affiliated Hospital of Sun Yat-sen University from 2017 to 2022. Adult patients (aged 18–75 years) undergoing whole LT with a graft procured from deceased donors were eligible for inclusion. Exclusion criteria were as follows: (1) patients younger than 18 years; (2) combined organ transplantation; (3) split LT; (4) LT with NMP; (5) half-circuit IFLT; (6) patients with a history of previous organ transplantation; and (7) LT cases with missing data that prevented the completion of the study. Upon organ allocation, informed consent for surgery was obtained from each patient before LT. The study was approved by the Institutional Review Boards of The First Affiliated Hospital, Sun Yat-sen University, which waived the need for written informed consent for research. (Approval number: [2024]286). All research procedures complied with the Declaration of Helsinki. The study cohort was divided into two groups based on the surgical method: the IFLT group and the CLT group.

### Study endpoints

The primary endpoint was the incidence of AKI with varying severities following LT. The secondary endpoints included the impact of IFLT on patient survival and related complications. Relevant indicators included the requirement of RRT within 7 days after LT, serum alanine transaminase (ALT) and aspartate aminotransferase (AST) levels within 7 days after LT, the incidence of early allograft dysfunction (EAD), in-hospital mortality, length of hospital stay after LT, length of intensive care unit (ICU) stay after LT, time on ventilator, 1-month patient survival, and 3-month patient survival.

### Definitions

AKI was defined and staged according to the 2012 Kidney Disease: Improving Global Outcomes criteria (KDIGO).^20^ AKI was diagnosed if any of the following criteria were met: (1) an increase in serum creatinine of more than 26.5 μmol/L (0.3 mg/dl) within 48 h; (2) a serum creatinine increase of more than 1.5 times the baseline, either confirmed or presumed to have occurred within 7 days; or (3) urine output of less than 0.5 ml/(kg·h) for more than 6 h. According to KDIGO, AKI was staged as follows:

Stage-1 AKI: a serum creatinine increase by 1.5–1.9 times the baseline or an absolute increase of more than 26.5 μmol/L (0.3 mg/dl), or urine output <0.5 ml/(kg·h) for 6–12 h.

Stage-2 AKI: a serum creatinine increase by 2.0–2.9 times the baseline, or urine output <0.5 ml/(kg·h) for ≥12 h.

Stage-3 AKI: a serum creatinine increase by ≥3 times the baseline, an absolute serum creatinine of ≥353.6 μmol/L (4.0 mg/dl), or initiation of RRT, urine output <0.3ml/(kg·h) for ≥24 h, or anuria lasting ≥12 h.

Transient AKI refers to an AKI episode that resolves within 48 h. Persistent AKI refers to an AKI episode that lasts longer than 48 h.

EAD was defined according to the criteria proposed by Olthoff *et al.*^21^ (Table S1). ECD livers were defined based on the criteria proposed by the German Federal Medical Association (Bundesärztekammer).^22^^,^^23^ The recipient's preoperative glomerular filtration rate (GFR) was calculated using the equation developed by the Modification of Diet in Renal Disease Study Group (MDRD)^24^:

$$\text{GFR}=186\times {\left(\text{Scr}\;\left[\frac{\mu \text{mol}}{L}\right]\right)}^{–1.154}✕{\text{age}}^{–0.203}\times 0.742\;\left(\text{female}\right)$$

### Ischemia-free liver transplantation

Fig. S1 provides a schematic overview of the IFLT procedure, illustrating its key steps and technical setup. IFLT aims to ensure uninterrupted blood flow throughout the LT process.^14^^,^^18^ Before liver retrieval, ∼1.2 L of leukocyte-depleted red blood cells (RBCs) and 1.2 L of succinylated gelatin (supplemented with heparin, magnesium sulphate, calcium chloride, amino acids, etc.) were preloaded into the NMP device. After the donor liver was prepared *in situ*, a 12Fr catheter was inserted into either the splenic artery or the gastroduodenal artery without interrupting the blood supply to the liver. A 32Fr catheter was placed in the inferior vena cava of the liver and connected to the organ chamber of the NMP device. Once the donor's transplant vessel (right external iliac vein) was bridged to the portal vein, a 24Fr catheter was inserted into the portal vein via the transplant vessel and connected to the portal vein conduit of the device. Then, the arterial catheter was connected to the arterial conduit of the device. After establishing the NMP circuit of the machine with the *in sit*u liver, the liver was retrieved and moved into the organ chamber of the machine for *ex situ* perfusion.

The machine continuously performed NMP on the isolated liver, primarily to both protect the graft from IRI and assess the quality of the transplant. The liver was perfused on the machine for 2–9 h, with the duration primarily dependent on the progress of the recipient's surgery. Following removal of the diseased liver, the donor liver was transferred from the organ chamber into the recipient's abdominal cavity. During the organ implantation process, the device continued to perfuse the liver, ensuring uninterrupted blood flow to the graft while performing the anastomoses of the portal vein and hepatic artery. After the vascular reconstruction of the liver was completed, NMP was discontinued and all catheters were removed.

### Statistical analysis

This study primarily used SPSS statistical software version 26.0 (IBM Corp., Armonk, NY, USA) for data processing and analysis. Continuous variables are presented as medians (IQR), whereas categorical variables are expressed as numbers (percentages). The normality of continuous variables was assessed using the Kolmogorov-Smirnov test. Normally distributed continuous variables were analyzed using Student's *t* test, whereas non-normally distributed continuous variables were analyzed using the Mann-Whitney *U* test. Categorical variables were compared using Pearson chi-squared test or Fisher's exact test. PSM, which helped balance baseline characteristics between the CLT group and the IFLT group, was conducted as described by Rubin to achieve an ‘approximate randomization’ effect.^25^ Patients were matched in a 1:1 ratio using propensity scores with a caliper width of 0.2. Kaplan-Meier analysis and the log-rank test were used to compare the survival rates. Multivariable logistic regression models were used to identify risk factors for post-transplant kidney function impairment. All analyses were deemed statistically significant at a two-sided *p* <0.05.

## Results

**Fig. 1:**
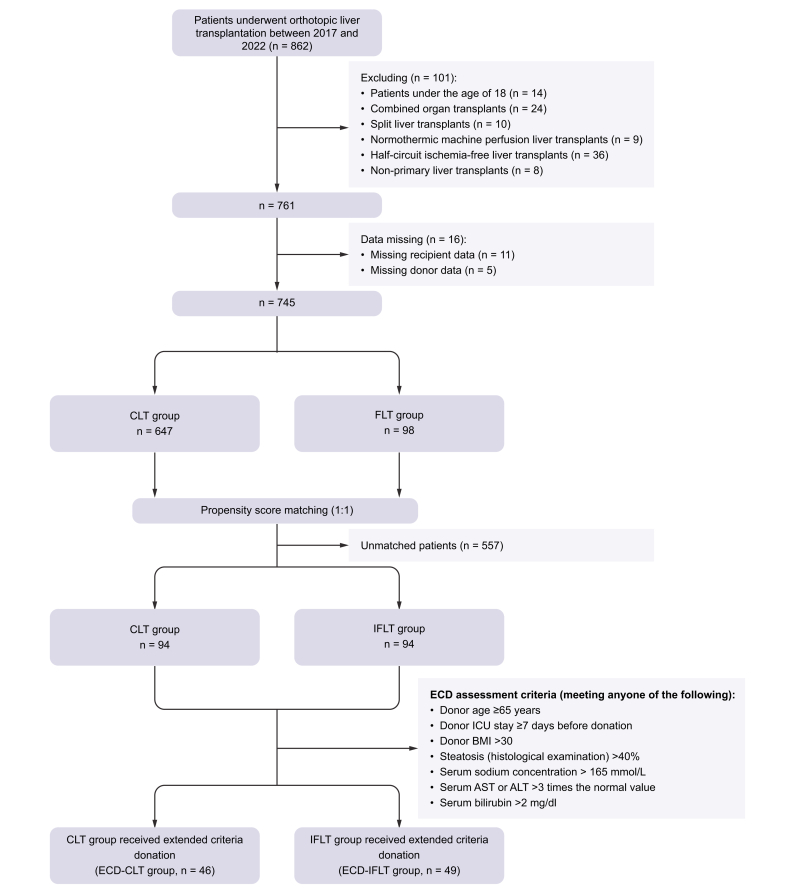
Flow diagram of the screening, enrollment, and allocation of patients. ALT, alanine transaminase; AST, aspartate aminotransferase; CLT, conventional liver transplantation; ECD, extended criteria donor; ICU, intensive care unit; IFLT, ischemia-free liver transplantation; PSM, propensity score matching.

In total, 745 patients were included in the final cohort, of which 98 underwent IFLT (Fig. 1). Before PSM, significant differences were observed in recipient age, incidence of ascites, hepatic encephalopathy (HE), hepatocellular carcinoma (HCC), donor age, donor sex, donor liver steatosis percentage, operation time, the intraoperative RBC and fresh-frozen plasma (FFP) usage, and the percentage of bicaval LT between the groups (Table 1). PSM created 94 pairs of patients. After PSM, the matching variables were well balanced, demonstrating an effective matching process (Table 1; Fig. S2).

**Table 1:** Baseline characteristics of patients between CLT group and IFLT group.

Baseline characteristics	Entire cohort		*p* value	PSM cohort		*p* value
	CLT (n = 647)	IFLT (n = 98)		CLT (n = 94)	IFLT (n = 94)	
**General**						
Age (years)^†^	51 (43–59)	52 (44–63)	**0.021**	53 (45–61)	52 (44–62)	0.887
Male	565 (87.3%)	87 (88.8%)	0.686	79 (84.0%)	84 (89.4%)	0.283
BMI (kg/m^2^)^†^	23.2 (20.8–24.8)	22.9 (21.5–24.9)	0.382	23.6 (21.4–25.5)	22.8 (21.5–25.0)	0.774
MELD score^†^	13 (9–22)	12 (9-–19)	0.865	14 (8–22)	12 (9–19)	0.935
Diabetes	94 (14.5%)	19 (19.4%)	0.211	17 (18.1%)	19 (20.2%)	0.711
HCC	370 (57.2%)	45 (45.9%)	**0.036**	36 (38.3%)	44 (46.8%)	0.238
Cirrhosis∗	145 (22.4%)	43 (43.9%)	**<0.001**	30 (31.9%)	40 (42.6%)	0.331

**Kidney function indicators**						
Preoperative RRT	34 (5.3%)	5 (5.1%)	0.949	6 (6.4%)	5 (5.3%)	0.756
Baseline creatinine (μmol/L)^†^	70 (59–84)	71 (58–86)	0.584	70 (58–83)	71 (58–85)	0.407
Baseline BUN (mmol/L)^†^	4.6 (3.7–6.4)	5.0 (3.9-–6.2)	0.355	5.0 (3.9–6.5)	4.9 (3.9–6.1)	0.809
MDRD4-EGFR (ml/[min × 1.73 m^2^])^†^	106.7 (87.5–131.2)	107.0 (81.1–128.2)	0.562	103.8 (88.2–131.7)	107.0 (81.7–129.1)	0.840

**Decompensated manifestations**						
Ascites			**0.001**			0.077
No	357 (55.2%)	35 (35.7%)		49 (52.1%)	34 (36.2%)	
Mild	168 (26.0%)	41 (41.8%)		27 (28.7%)	39 (41.5%)	
Severe	122 (18.9%)	22 (22.4%)		18 (19.1%)	21 (22.3%)	
HE			**0.008**			0.060
No	566 (87.5%)	78 (79.6%)		81 (86.2%)	76 (80.9%)	
Stage I/II	46 (7.1%)	16 (16.3%)		6 (6.4%)	15 (16.0%)	
Stage III/IV	35 (5.4%)	4 (4.1%)		7 (7.4%)	3 (3.2%)	

**Donor features**						
Age (years)^†^	39 (23–47)	45 (31–52)	**<0.001**	43 (31–51)	45 (31–51)	0.903
Male	148 (22.9%)	41 (41.8%)	**<0.001**	25 (26.6%)	37 (39.4%)	0.063
BMI (kg/m^2^)^†^	22.3 (20.4–24.1)	22.0 (19.0–23.9)	0.121	22.0 (19.9–23.7)	22.2 (19.5–24.0)	0.736
AST (U/L)^†^	51 (35–91)	48 (33–62)	0.295	62 (35–104)	60 (36–97)	0.789
Donor liver steatosis	218 (33.7%)	47 (48.0%)	**0.006**	35(37.2%)	43 (45.7%)	0.236
Mild (steatosis <30%)	201(31.1%)	42 (42.9%)		35 (37.2%)	38 (40.4%)	
Moderate (steatosis <60%)	9 (1.4%)	3 (3.1%)		0 (0%)	3 (3.2%)	
Severe (steatosis ≥60%)	8 (1.2%)	2 (2.0%)		0 (0%)	2 (2.1%)	

**Operation features**						
Anhepatic period (min)^†^	55 (43–65)	53 (45–62)	0.062	56 (42–67)	54 (45–62)	0.718
Operation time (h)^†^	7.8 (6.7–9.0)	6.5 (5.9–7.4)	**<0.001**	7.8 (6.6–9.1)	6.5 (5.8–7.4)	**<0.001**
RBC (u)^†^	5.0 (3.3–9.0)	4.0 (2.0–7.5)	**0.004**	5.0 (3.0–7.0)	4.0 (3.0–8.0)	0.301
FFP (ml)^†^	1,600 (1,000–2,200)	1,000 (625–1,375)	**<0.001**	1,550 (1,100–1,963)	1,000 (672–1,375)	**<0.001**
Piggyback LT	60 (9.3%)	36 (36.7%)	**<0.001**	58 (61.7%)	35 (37.2%)	**0.001**
Bicaval LT	586 (90.6%)	62 (63.3%)		36 (38.2%)	59 (62.8%)	

∗Cirrhosis: refers to decompensated cirrhosis related LT, excluding HCC. Values in parentheses are percentages unless indicated otherwise; values are ^†^median (IQR).

Bold indicates that the p-value is less than 0.05, signifying a statistically significant difference.

AST, aspartate aminotransferase; CLT, conventional liver transplantation; FFP, fresh-frozen plasma; HCC, hepatocellular carcinoma; HE, hepatic encephalopathy; IFLT, ischemia-free liver transplantation; LT, liver transplantation; MELD, Model for End-Stage Liver Disease; MDRD4-EGFR, estimated glomerular filtration rate calculated using the equation of the Modification of Diet in Renal Disease Study Group; RBC, red blood cells; RRT, renal replacement therapy.

### Comparison of postoperative renal function between CLT and IFLT groups

**Fig. 2:**
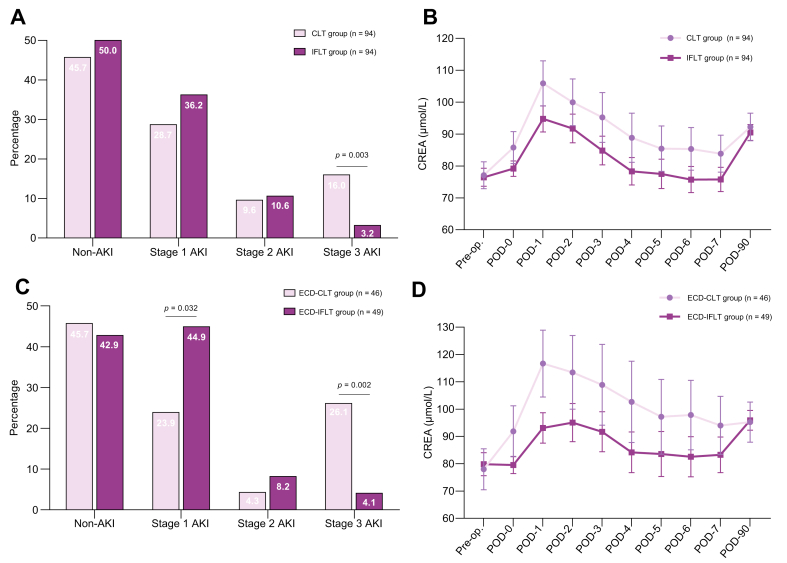
Comparison of post-LT renal function between the CLT and IFLT groups. (A) Differences in AKI severity between the CLT and IFLT groups of the PSM cohort. Levels of significance: non-AKI, *p* = 0.011; Stage-1 AKI, *p* = 0.276; Stage-2 AKI, *p* = 0.809; Stage-3 AKI, *p* = 0.003 (Pearson chi-squared test). (B) Creatinine levels in the CLT *vs.* IFLT group of the PSM cohort. Levels of significance: all *p* >0.05 (Mann-Whitney *U* test). (C) Differences in AKI severity between the CLT and IFLT groups of the ECD subgroup. Levels of significance: non-AKI, *p* = 0.784; Stage-1 AKI, *p* = 0.032; Stage-2 AKI, *p* = 0.678; Stage-3 AKI, *p* = 0.002 (Pearson chi-squared test). (D) Creatinine levels in the CLT *vs.* IFLT group of the ECD subgroup. Levels of significance: except for POD-1, all *p* >0.05 (Mann-Whitney *U* test). AKI, acute kidney injury; CLT, conventional liver transplantation; CREA, creatinine; ECD, extended criteria donor; IFLT, ischemia-free liver transplantation; POD, postoperative day; PSM, propensity score matching.

The overall incidence of AKI in the IFLT group (50.0%) was lower than in the CLT group (54.3%), although this difference was not statistically significant (*p* = 0.559) (Table 2 and Fig. 2A). Importantly, the incidence of Stage-3 AKI in the IFLT group was lower than that in the CLT group (IFLT 3.2% *vs.* CLT 16.0%, *p* = 0.003), whereas that of Stage-2 AKI was comparable between the groups (IFLT 10.6% *vs.* CLT 9.6%, *p* = 0.809).

**Table 2:** Comparison of postoperative renal function between the IFLT and CLT groups after matching.

Measurement	CLT (n = 94)	IFLT (n = 94)	*p* value
Acute kidney injury	51 (54.3%)	47 (50.0%)	0.559
Stage 1	27 (28.7%)	34 (36.2%)	0.276
Stage 2	9 (9.6%)	10 (10.6%)	0.809
Stage 3	15 (16.0%)	3 (3.2%)	0.003
Acute kidney injury severity			
Mild†	27 (28.7%)	34 (36.2%)	0.276
Severe‡	24 (25.5%)	13 (13.8%)	0.044
Acute kidney injury duration			
Transient§	28 (29.8%)	39 (41.5%)	0.094
Persistent¶	23 (24.5%)	8 (8.5%)	0.003
Postoperative renal replacement therapy	12 (12.8%)	3 (3.2%)	0.015
Initial∗∗	8 (8.5%)	2 (2.1%)	0.051
Persistent‡‡	4(4.3%)	1 (1.1%)	0.368
Preoperative renal replacement therapy	6 (6.4%)	5 (5.3%)	0.756
RRT cessation§§	2 (2.1%)	4 (4.3%)	0.682
Percentage of RRT cessation within POD 7¶¶	2/6 (33.3%)	4/5 (80.0%)	0.242
Creatinine level			
Peak creatinine within POD 7 (μmol/L)	94 (74–131)	92 (78–120)	0.927
Peak creatinine within POD 7/baseline creatinine∗	1.3 (1.0–1.8)	1.3 (1.1–1.7)	0.985
Change in serum creatinine (μmol/L)∗^,^∗∗	19 (2–47)	21 (6–41)	0.811

Values in parentheses are percentages unless indicated otherwise.

AKI, acute kidney injury; CLT, conventional liver transplantation; IFLT, ischemia-free liver transplantation; POD, postoperative day; RRT, renal replacement therapy; SAKI, severe acute kidney injury.

∗ Median (IQR).

† Mild AKI: refers to Stage-1 AKI.

‡ Severe AKI: including Stage-2 AKI and Stage-3 AKI.

§ Transient AKI: AKI lasting <48 h.

¶ Persistent AKI: AKI lasting ≥48 h.

∗∗ Initial RRT: refers to patients who did not receive RRT before LT and received RRT for the first time within 7 days postoperatively.

‡‡ Persistent RRT: refers to the condition where RRT is required both preoperatively and postoperatively.

§§ RRT cessation: refers to patients who required RRT before LT but no longer needed RRT within 7 days postoperatively. Percentage of RRT cessation within 7 days postoperatively = number of RRT cessation/number of patients who received RRT treatment before surgery.

¶¶ Change in serum creatinine = peak creatinine within 7 days postoperatively – baseline creatinine before surgery.

Patients with Stage-1 AKI showed no significant difference in 3-month patient survival compared with those without AKI in the entire cohort (*p* = 0.418) (Fig. S3B). By contrast, patients with Stage-2 and Stage-3 AKI experienced significantly lower 3-month survival rates (all *p <*0.001). Among the patients with Stage-3 AKI, the proportion of persistent AKI was the highest (83.3%); 10 of these patients (66.7%) had not recovered 1 month after surgery (Fig. S3C). Notably, the incidence of persistent AKI was significantly lower in the IFLT group compared with the CLT group (IFLT 8.5% *vs.* CLT 24.5%, *p* = 0.003; Table 2).

In terms of the need for postoperative RRT, the postoperative RRT proportion of the IFLT group was lower than that of the CLT group (IFLT 3.2% *vs.* CLT 12.8%, *p* = 0.015). Among the patients who required postoperative RRT, two patients in the IFLT group and eight in the CLT group did no require preoperative RRT (initial RRT rate: IFLT 2.1% *vs.* CLT 8.5%, *p* = 0.051).

In patients who required preoperative RRT, four out of five patients in the IFLT group no longer required RRT within 7 days post operation, whereas only two out of six patients in the CLT group no longer required RRT within 7 days post operation (cessation rate: IFLT 80.0% *vs.* CLT 33.3%, *p* = 0.242). Within 7 days post operation, the daily creatinine levels of the IFLT group were lower than in the CLT group, although nonsignificantly so (*p* >0.05) (Fig. 2B).

### Comparison of postoperative prognosis between CLT and IFLT groups

Only eight patients (8.5%) in the IFLT group experienced EAD, compared with 42 patients (44.7%) in the CLT group (*p* <0.001) (Table 3). The peak AST and ALT levels within 7 days post operation were significantly lower in the IFLT group than in the CLT group (*p* <0.001). None of the patients in the IFLT group experienced in-hospital death, whereas the in-hospital mortality rate of the CLT group was as high as 9.6% (*p* = 0.003). The 3-month patient survival was higher in the IFLT group compared with the CLT group, but the difference was nonsignificant (94.7% *vs.* 91.5%, *p* = 0.377) (Table 3; Fig. S4).

**Table 3:** Comparison of survival and postoperative complications between the IFLT and CLT groups after matching.

Measurement	CLT (n = 94)	IFLT (n = 94)	*p* value
**Postoperative status**			
Peak AST within POD 7 (U/L)∗	1,268 (482–2,927)	439 (242–922)	**<0.001**
Peak ALT within POD 7 (U/L)∗	535 (251–1,104)	183 (118–406)	**<0.001**
EAD	42 (44.7%)	8 (8.5%)	**<0.001**
In-hospital death	9 (9.6%)	0 (0%)	**0.003**
Length of hospital stay after LT (d)∗	23 (16–31)	19 (12–34)	0.199
Length of ICU stay after LT (h)∗	36 (22–96)	35 (20–59)	0.311
Time on ventilator (h)∗	18.5 (10.6–56.1)	16.0 (12.0–34.0)	0.576
Patient death within 3 months	8 (8.5%)	5 (5.3%)	0.377

**Cause of patient death within 3 months**			
Primary non-function	1 (1.1%)	0(0%)	–
Multiple organ failure	2 (2.1%)	2 (2.1%)	–
Liver failure	2 (2.1%)	0 (0%)	–
Severe infection	3 (3.2%)	2 (2.1%)	–
Pulmonary embolism	0 (0%)	1 (1.1%)	–

Values in parentheses are percentages unless indicated otherwise. Bold indicates that the p-value is less than 0.05, signifying a statistically significant difference.

ALT, alanine aminotransferase; AST, aspartate aminotransferase; CLT, conventional liver transplantation; EAD, early allograft dysfunction; ICU, intensive care unit; IFLT, ischemia-free liver transplantation; LT, liver transplantation; POD, postoperative day.

∗ Median (IQR).

The occurrence of SAKI was significantly associated with a worse prognosis among patients undergoing CLT (Table S3). However, unlike the CLT group, there were no significant differences in postoperative liver function, complications and survival between the IFLT-nonSAKI group and IFLT-SAKI group. The CLT-SAKI group had the poorest 3-month survival (70.8%), whereas patients with SAKI who underwent IFLT (IFLT-SAKI group) had an improved 3-month survival (92.3%), although the difference was not statistically significant (*p* = 0.140) (Fig. S5). Three-month survival did not differ significantly between the IFLT-SAKI group and the IFLT-nonSAKI group (92.3% *vs.* 95.1%, *p* = 0.682) or the CLT-nonSAKI group (92.3% *vs.* 98.6%, *p* = 0.184).

### Analysis of ECD subgroups

In the PSM cohort, ECD livers were received in 49 IFLT patients and 46 CLT patients. The ECD-IFLT group had shorter surgery time (*p* <0.001), less intraoperative FFP transfusion (*p* <0.001), a higher proportion of male donors (*p* = 0.035), and a lower proportion of controlled cardiac-death donors (*p* <0.001) compared with the ECD-CLT group (Table S4). Aside from these differences, there were no other significant disparities between the groups.

The AKI incidence in the ECD-IFLT group (57.1%) was similar to that in the CLT group (54.3%) (*p* = 0.784) (Fig. 2C,D; Table S5). The tendency of IFLT patients to develop less severe AKI compared with CLT patients persisted within the ECD subgroup. The incidence of Stage-3 AKI decreased in the ECD-IFLT group compared with the ECD-CLT group, (*p* = 0.002). The number of patients with persistent AKI in the ECD-CLT group was higher than in the ECD-IFLT group (*p* = 0.006), whereas those requiring RRT was lower in the ECD-IFLT group, but the difference was not significant (*p* = 0.190). Within 1 week after LT, the daily creatinine levels in the ECD-IFLT group were lower than in the ECD-CLT group, but the difference was not significant (Fig. 2D). These results again demonstrated that AKI among patients who underwent IFLT was mostly mild renal injury, whereas patients in the CLT group experienced more severe renal injury (SAKI: ECD-IFLT 12.3% *vs.* ECD-CLT 30.4%, *p* = 0.030). Moreover, this difference was more pronounced in the ECD subgroup. The SAKI reduction rate of ECD subgroup was 18.1% compared with 11.7% in the PSM cohort.

### Multivariate regression analysis of renal injury after liver transplantation

Based on clinical experience and the aim of the study, the multivariate logistic regression analysis included surgical, recipient, donor, and kidney function-related parameters. After adjusting for the above factors, IFLT remained a protective factor against the development of post-LT Stage-3 AKI in transplant patients (odds ratio 0.166; 95% CI, 0.028–0.979; *p* = 0.047) (Table 4), as did avoiding the use of ECD.

**Table 4:** Regression analysis of risk factors for postoperative Stage-3 AKI.

Measurements	Multivariate regression analysis∗		
	Odds ratio	95% CI	*p* value
**Surgery-related parameters**			
IFLT	0.166	0.028–0.979	**0.047**
Bicaval LT	1.316	0.346–5.009	0.687
Intraoperative FFP usage (ml)	1.000	0.999–1.001	0.573
Intraoperative RBC usage (u)	1.132	0.954–1.344	0.157
Operation time (h)	1.151	0.758–1.747	0.510
Anhepatic period (min)	1.018	0.978–1.059	0.388

**Recipient-related parameters**			
Recipient age (years)	0.958	0.890–1.031	0.254
Recipient BMI (kg/m^2^)	1.133	0.930–1.382	0.216
Preoperative diabetes	1.647	0.294–9.218	0.570

**Donor-related parameters**			
Donor age (years)	1.009	0.954–1.066	0.764
Donor BMI (kg/m^2^)	1.132	0.903–1.419	0.283
ECD liver	10.796	1.914–60.895	**0.007**
Steatosis donor liver	1.456	0.390–5.440	0.576
Cardiac-death donor liver	1.548	0.095–25.326	0.759
Warm ischemia time (min)	0.960	0.745–1.235	0.749

**kidney function-related parameters**			
Preoperative RRT	6.091	0.650–57.052	0.113
Preoperative BUN (mmol/L)	0.984	0.753–1.285	0.905
MDRD4-EGFR (ml/[min × 1.73 m^2^])	0.987	0.966–1.009	0.236

Values in parentheses are CI 95% unless indicated otherwise. Bold indicates that the p-value is less than 0.05, signifying a statistically significant difference.

BUN, blood urea nitrogen; ECD, extended criteria donor; FFP, fresh frozen plasma; IFLT, ischemia-free liver transplantation; MDRD4-EGFR, estimated glomerular filtration rate calculated using equation of the Modification of Diet in Renal Disease Study Group; RBC, red blood cells; RRT, renal replacement therapy.

∗ Multivariate logistic regression analysis was performed, incorporating surgery-related parameters (IFLT, operation time, intraoperative FFP usage, intraoperative RBC usage, bicaval LT, and anhepatic period), recipient-related parameters (age, BMI, and presence of diabetes), donor-related parameters (age, BMI, ECD donor, steatosis donor liver, cardiac-death donor, and warm ischemia time), as well as kidney function-related parameters (preoperative BUN levels, preoperative MDRD4-EGFR, and preoperative RRT).

## Discussion

In this study, we used a post hoc randomization method PSM to systematically investigate the protective effect of IFLT on post-LT AKI. IFLT was shown to be an effective LT approach to protect the renal function of patients after LT. Our analysis demonstrated that IFLT significantly reduced the incidence of postoperative SAKI, Stage-3 AKI, and RRT in patients after LT. Importantly, this protective effect was even more pronounced among patients receiving ECD livers.

In 2021, Wong *et al.* reported that Stage-2 and Stage-3 AKI had a more severe impact on patient prognosis compared with Stage-1 AKI.^26^ Our study had similar findings: there was no significant difference in 3-month survival in patients with Stage-1 AKI compared with those without AKI, whereas patients with Stage-2 or Stage-3 AKI had a significantly reduced 3-month survival in the entire cohort (Fig. S3B). Stage-1 AKI predominantly involved transient kidney damage, whereas Stage-2 and Stage-3 AKI tended to result in persistent kidney impairment (Fig. S3C). Therefore, we focused on exploring whether IFLT impacts SAKI, namely Stage-2 and Stage-3 AKI. Effective pharmacological treatments for SAKI are still lacking and, thus, the prevention of AKI after LT is emphasized. Excitingly, we found that IFLT reduced the severity of kidney injury after LT (SAKI: IFLT 13.8% *vs.* CLT 25.5%, *p* = 0.044). Most of cases of AKI in the IFLT group were mild and transient, with kidney function recovering within 48 h after surgery.

We also analyzed the potential reasons for the protective effect of IFLT on post-transplant AKI. IRI is considered one of the key surgical factors contributing to AKI,^10^^,^^11^ whereby it triggers a systemic inflammatory response, which leads to damage of renal tubular epithelial and vascular endothelial cells, as well as hemodynamic fluctuations and renal hypoperfusion. IRI is an inherent drawback in organ transplantation, and current clinical strategies for treating IRI show limited efficacy. In recent years, machine perfusion (MP) has been increasingly recognized as an important strategy to protect donor livers from IRI.[27], [28], [29], [30] Furthermore, the flushing effect of MP can reduce the entry of inflammatory factors into the body during graft reperfusion, alleviate vasospasm, stabilize the hemodynamics, and reduce the occurrence of renal hypoperfusion. This suggests a potential role for MP in protecting post-transplant kidney function. In a 2021 study, Czigany *et al.* compared hypothermic oxygenated machine perfusion (HOPE) with CLT in donation after brain death (DBD) LT and showed that HOPE helped reduce peak creatinine levels within 7 days after LT (CLT 2.28, 1.33–3.47 mg/dl *vs.* HOPE 1.45, 1.07–2.20 mg/dl, *p* = 0.071) and the RRT occurrence within 90 days after LT (CLT 39% *vs.* HOPE 22%, *p* = 0.337).^23^ In 2022, Gaurav *et al.* conducted a retrospective comparative analysis of CLT, normothermic regional perfusion (NRP), and normothermic machine perfusion (NMP) in donation after cardiac death (DCD) LT, and suggested the renal protective effect of MP (AKI incidence: CLT 55% *vs.* NRP 39% *vs.* NMP 40%, *p* = 0.08), with NRP significantly reducing the postoperative peak/baseline creatinine ratio (*p* = 0.02).^31^ However, existing reports did not show significant differences in the protective effects of HOPE and NMP on postoperative AKI compared with CLT. This might be because these MP approaches did not completely eliminate IRI. NMP technology ensures an organ blood supply during *ex vivo* organ preservation but subjects the organ to repeat IRI during organ procurement and implantation. By contrast, HOPE uses cooled oxygenated perfusate to perfuse organs, allowing tissue energy reserves to be restored before reperfusion and thereby reducing IRI, but it does not eliminate it entirely.

IFLT, which is based on NMP technology, aims to continuously supply blood to the liver throughout the organ transplantation process,^14^ an approach that completely avoids IRI.^15^ The post-transplantation condition of both the transplant liver and the patient indirectly reflects this conclusion. We found that only eight patients (8.5%) in the IFLT group experienced EAD, compared with 42 patients (44.7%) in the CLT group. In the CLT group, the CLT-SAKI group had poorer prognosis compared with the CLT-nonSAKI group. However, in the IFLT group, there were no significant differences in postoperative graft function, postoperative complications, in-hospital mortality rate, and postoperative survival between the IFLT-nonSAKI and the IFLT-SAKI groups. This further indirectly reflects the contribution of IFLT to reducing IRI.

Although the use of ECD organs significantly expands the donor pool,^7^ these predamaged or lower-quality organs have less tolerance to IRI, leading to a significantly increased incidence of AKI after marginal LT.^8^^,^^9^ Interestingly, the trend of worse postoperative renal function was not seen among patients underwent IFLT with ECD grafts. The trend of less severe AKI in the IFLT group compared with the CLT group was also present within the ECD subgroup, a difference that was even more pronounced in the ECD subgroup. This result further supports the conclusion that IFLT helps alleviate IRI and consequently protects post-LT kidney function.

## Conclusions

This study demonstrated that IFLT is an effective surgical approach to protect the renal function of patients after LT. Importantly, this protective effect was even more pronounced among patients receiving ECD livers.

## Abbreviations

AKI, acute kidney injury; ALT, alanine transaminase; AST, aspartate aminotransferase; BUN, blood urea nitrogen; CLT, conventional liver transplantation; CREA, creatinine; DBD, donation after brain death; DCD, donation after cardiac death; EAD, early allograft dysfunction; ECD, extended criteria donor; FFP, fresh-frozen plasma; GFR, glomerular filtration rate; HCC, hepatocellular carcinoma; HE, hepatic encephalopathy; HOPE, hypothermic oxygenated machine perfusion; ICU, intensive care unit; IFLT, ischemia-free liver transplantation; IRI, ischemia-reperfusion injury; KDIGO, Kidney Disease: Improving Global Outcomes criteria; LT, liver transplantation; MDRD, Modification of Diet in Renal Disease Study Group; MP, machine perfusion; NMP, normothermic machine perfusion; NRP, normothermic regional perfusion; POD, postoperative day; PSM, propensity score matching; RBC, red blood cell; RRT, renal replacement therapy; SAKI, severe acute kidney injury; SCS, static cold storage; SIRS, systemic inflammatory response syndrome.

## Financial support

This work was supported by grants as follows: National Natural Science Foundation of China (82470687, 8227031964, and 822706886), National Key Research and Development Plan Disruptive Technological Innovation (2023YFF1501202), Colin New Star of Sun Yat-Sen University (R08027), Colin New Talents Project of Sun Yat-Sen University (R07015), and the Guangdong Science and Technology Innovation Strategy (pdjh2022b0010 and pdjh2023a0002).

## Authors’ contributions

Study design: QZ, YN, JH, XH. Data collection: JH, YN, MQ, YT, ZL. Data analysis: JH, YN, MQ, YL, JD. Manuscript writing: JH, QZ, JD, YL, ZG.

## Data availability statement

The data involved in this study can be obtained by contacting the corresponding authors.

## Conflicts of interest

The authors have no conflicts of interest to disclose.

Please refer to the accompanying ICMJE disclosure forms for further details.
