# Supplementary material for: Protective value of ischemia-free liver transplantation on post-transplant acute kidney injury
Source: JHEP Rep. 2025 Jan 29;7(4):101339. doi: 10.1016/j.jhepr.2025.101339 (PMC11986513; doi:10.1016/j.jhepr.2025.101339)
Supplement: Multimedia component 1 [file mmc1.pdf]

The protective value of ischemia-free liver transplantation on post-transplant acute kidney injury

Qiang Zhao, Jinbo Huang, Meiting Qin, Yunhua Tang, Zhiying Liu, Yefu Li, Zhiyong Guo, Jia Dan, Yu Nie, Xiaoshun He

Table of contents

Fig. S1..2

Fig. S2..3

Fig. S3..4

Fig. S4..5

Fig. S5..6

Table S1..7

Table S2..9

Table S3..11

Table S4..12

Table S5..14

Fig. S1 Graphic rendering of the ischemia-free liver transplantation procedure.

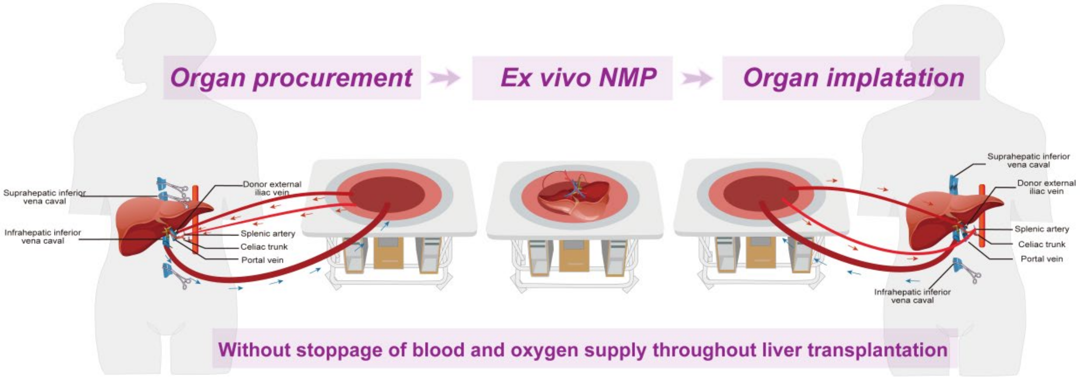

Fig. S2 Matching effectiveness among patients of the IFLT Group and CLT Group (Propensity Score Matching). Based on the clinical experience of transplant doctors and the primary objective of this study, 14 parameters including recipient characteristics (age, BMI, presence of hepatocellular carcinoma, MELD score), donor characteristics (age, BMI, serum AST levels), surgical factors (the time of anhepatic period, red blood cell transfusion volume, fresh frozen plasma transfusion volume), and renal function indicators (preoperative serum creatinine level, preoperative BUN level, preoperative eGFR, preoperative RRT) were included in the PSM. (A) Scatter plot depicting the distribution of propensity scores of each patient before and after matching. (B) Bar chart illustrating the distribution of propensity scores of each group before and after matching.

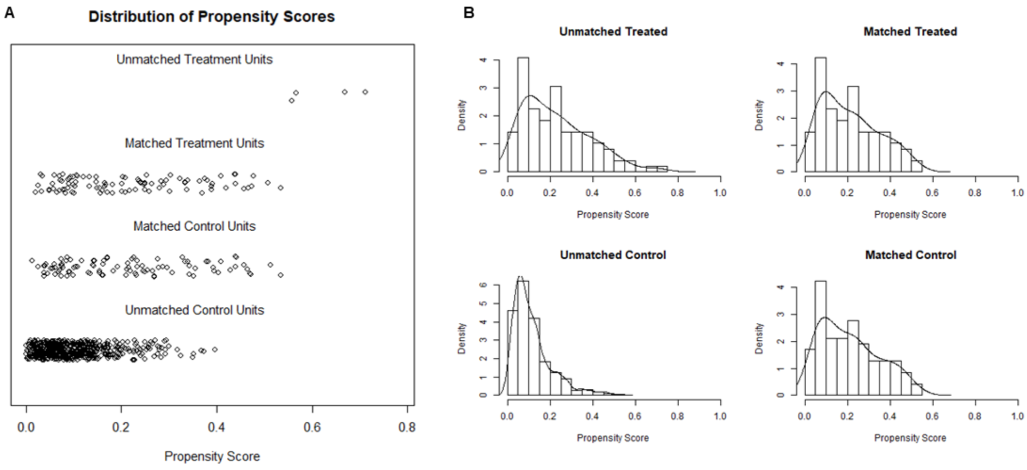

Fig. S3 Different severity AKI and their impact on patient survival. (A) Comparison of 3-month patient survival between patients who developed AKI and those who did not in the entire cohort. Levels of significance: $p < 0.001$ (Log-rank test). (B) Comparison of 3-month patient survival among patients with different severity AKI in the entire cohort. The P-values indicate respectively whether there are significant differences in comparison of 3-month patient survival between non-AKI group and the Stage-1 AKI group, Stage-2 AKI group or Stage-3 AKI group. Levels of significance: all $p < 0.001$ (Log-rank test). (C) Comparison of the characteristics of different severity AKI in the PSM cohort.

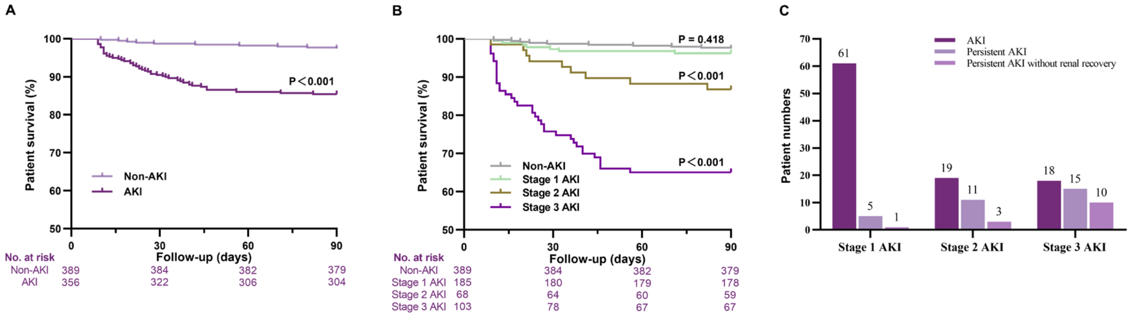

Fig. S4 The three-month patient survival curve of the IFLT group and the CLT group. Levels of significance: p = 0.377 (Log-rank test).

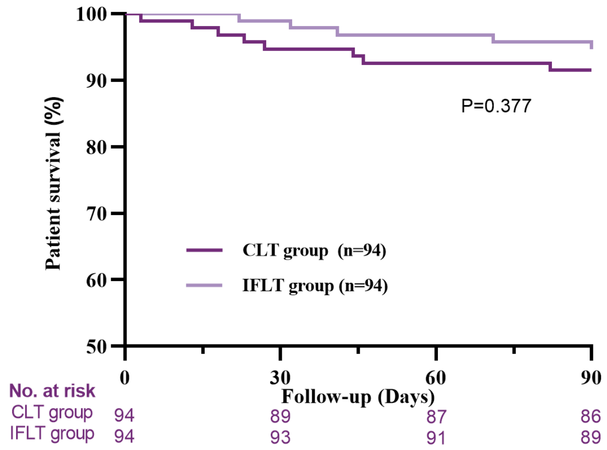

Fig. S5 3-Month patient survival curves for IFLT-SAKI group, IFLT-nonSAKI group, CLT-SAKI group and CLT-nonSAKI group. The P-values in the figure indicate respectively whether there is statistical significance in the comparison of 3-month patient survival between the CLT-nonSAKI group and the other three groups. Levels of significance: IFLT-SAKI group vs CLT-SAKI group, $P = 0.140$; IFLT-nonSAKI group vs CLT-SAKI group, $P < 0.001$; CLT-nonSAKI group vs CLT-SAKI group, $P < 0.001$. (Log-rank test).

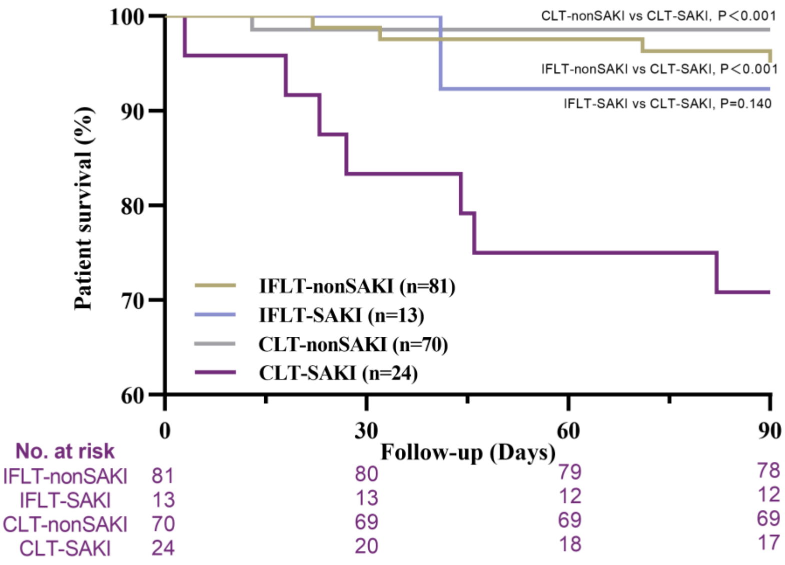

Table S1. Definition or evaluation criteria of the related parameters.

Parameters	Definition or evaluation criteria
**Early Allograft Dysfunction (EAD)**	EAD was diagnosed when meeting anyone of the followings:<br>(1) Serum AST or ALT levels greater than 2000U/L during the first week after transplantation<br>(2) International Normalized Ratio (INR) of 1.6 or higher on postoperative day 7<br>(3) Total bilirubin (Tbil) level of 10 mg/dL or higher on postoperative day 7
**Extended Criteria Donor (ECD)**	ECD was confirmed when meeting any of the followings:<br>(1) Donor age of 65 years or older<br>(2) Donor ICU stay of 7 days or longer before donation<br>(3) Donor BMI >30<br>(4) Steatosis (histological examination) > 40%<br>(5) Serum sodium concentration >165 mmol/L<br>(6) Serum AST or ALT >3 times the normal value<br>(7) Serum bilirubin >2 mg/dl
**Mild Acute Kidney Injury**	Refers to Stage-1 AKI
**Severe Acute Kidney Injury (sAKI)**	Both Stage-2 and Stage-3 AKI are classified as severe AKI
**Transient AKI**	AKI lasting < 48 hours
**Persistent AKI**	AKI lasting ⩾ 48 hours
**Initial RRT**	The situation of patients who did not receive RRT before LT and received RRT for the first time within 7 days postoperatively
**Persistent RRT**	Refers to the condition where RRT is required both preoperatively and postoperatively
**RRT cessation**	The situation of patients who required RRT before LT but no longer needed RRT within 7 days postoperatively
**Percentage of RRT cessation within 7 days postoperatively**	the number of patients who received RRT treatment before surgery but discontinued RRT treatment after surgery / the number of patients who received RRT treatment before surgery

**Change in serum creatinine**	the maximum creatinine level within 7 days postoperatively - the baseline creatinine level before surgery

Table S2. Baseline characteristics of liver transplant recipients, donors, and surgical procedures (n=745)

Baseline Parameters	Orthotopic liver transplantation（n=745）
**Recipient features**	
Age（years）	51（43-59）
Sex（male,%）	652（87.5%）
BMI (kg/m^2)	23.2（20.8-24.8）
HCC	415（55.7%）
Cirrhosis[#]	188（25.2%）
MELD Score	13（9-21）
Preoperative RRT（n,%）	39（5.2%）
Ascites（n,%）	
None	392（52.6%）
Mild	209（28.1%）
Severe	144（19.3%）
HE（n,%）	
No	644（86.5%）
Stage Ⅰ/Ⅱ	62（8.3%）
Stage Ⅲ/Ⅳ	39（5.2%）
Child stage（n,%）	
Stage A	281（37.7%）
Stage B	289（38.8%）
Stage C	175（23.5%）
**Donor features**	
Age（years）	40（25-48）
Sex（male,%）	189（25.4%）
BMI, kg/m^2	22.3（20.3-24.1）
Cause of Death (n,%)	
Trauma	360（48.3%）
Cerebrovascular Accident	252（33.8%）
Hypoxia	52（7.0%）
Others	81（10.9%）
Steatosis of the donor livers	265（35.6%）
Mild（steatosis<30%）	243（32.7%）
Moderate(steatosis<60%)	12（1.6%）

Severe(steatosis⩾60%)	10（1.3%）
Cardiac-death donor livers	141(18.9%)
Brain-death donor Livers	604(81.1%)
**Operation features**	
Anhepatic period（min）	55.0（44.0-65.0）
Operation time（h）	7.5（6.5-8.8）
RBC（u）	5.0（3.0-9.0）
FFP（ml）	1450.0（1000.0-2056.3）
Piggyback LT（n,%）	96（13.0%）
Orthotopic LT（n,%）	648（87.0%）

BMI, Body Mass Index；MELD, Model for End-Stage Liver Disease；RRT, Renal Replacement Therapy; HE, Hepatic Encephalopathy；RBC, Red Blood Cells；FFP, Fresh Frozen Plasma.

Cirrhosis#：refers to decompensated cirrhosis related LT, excluding HCC.

Table S3. Comparison of postoperative complications and survival between SAKI Group and Non-SAKI Group.

	CLT（n=94）		*P*	IFLT（n=94）		*P*
	**nonSAKI (n=70)**	**SAKI （n=24）**		**nonSAKI (n=81)**	**SAKI (n=13)**	
Peak AST within POD 7（U/L）	1201（476-2143）	1672（498-7978）	0.126	479（276-922）	341（160-863）	0.281
Peak ALT within POD 7（U/L）	531（253-871）	852（223-2160）	0.158	199（121-406）	146（103-380）	0.356
EAD（n,%）	25（35.7%）	17（70.8%）	**0.003**	5（6.2%）	3（23.1%）	0.078
In-hospital death（n,%）	1（1.4%）	8（33.3%）	**<0.001**	0（0%）	0（0%）	1.000
length of hospital stay after LT（d）	22（15-29）	27（20-43）	**0.044**	18（16-29）	23（18-45）	0.147
length of ICU stay after LT（h）	31.5（19.9-64.6）	146.5（35.8-391.0）	**<0.001**	35.0（20.0-59.0）	39.6（24.5-103.0）	0.298
Time on Ventilator（h）	15.0（10.3-35.6）	64.5（16.0-165.5）	**0.001**	16.0（12.0-34.1）	17.5（11.4-54.8）	0.842
1-month Patient death（n,%）	1（1.4%）	4（16.7%）	**0.004**	1（1.2%）	0（0%）	0.689
3-month Patient death（n,%）	1（1.4%）	7（29.2%）	**<0.001**	4（4.9%）	1（7.7%）	0.682

CLT, Conventional Liver Transplantation; IFLT, Ischemia-Free Liver Transplantation; SAKI, Severe AKI (including Stage-2 and Stage-3 AKI); nonSAKI, non-severe AKI (including patients with no AKI and Stage 1 AKI); AST, Aspartate Aminotransferase; ALT, Alanine Aminotransferase; EAD, Early Allograft Dysfunction; ICU, Intensive Care Unit.

Table S4. Comparison of baseline characteristics of patients between ECD-IFLT Group and ECD-CLT Group .

	ECD-CLT (n=46)	ECD-IFLT (n=49)	*P*
**Recipient features**			
**General condition**			
Age（years）	53（46-60）	53（47-63）	0.766
Sex（male,%）	40（87.0%）	45（91.8%）	0.516
BMI (kg/m^2)	23.6（21.4-25.6）	23.7（21.6-25.4）	0.569
MELD Score	12（8-21）	13（9-19）	0.561
Diabetes（n,%）	9（19.6%）	10（20.4%）	0.918
HCC（n,%）	22（47.8%）	25（51.0%）	0.756
**Kidney function indicators**			
Preoperative RRT（n,%）	3（6.5%）	3（6.1%）	0.631
Baseline creatinine（umol/L）	69（54-84）	72（61-89）	0.187
Baseline BUN（mmol/L）	5.0（4.1-6.2）	5.0（3.9-6.1）	0.685
MDRD4-EGFR（ml/(min*1.73m^2)）	102.8（88.7-140.3）	108.8（74.0-128.5）	0.404
**Donor features**			
Age（years）	43（29-50）	44（29-51）	0.920
Sex（male,%）	8（17.4%）	18（36.7%）	**0.035**
BMI (kg/m^2)	22.0（19.8-24.1）	22.5（20.3-24.0）	0.517
AST（U/L）	103（43-162）	83（52-134）	0.571
Donor liver steatosis	19（41.3%）	23（46.9%）	0.581
Mild（steatosis<30%）	19（41.3%）	20（40.8%）	
Moderate(steatosis<60%)	0（0%）	2（4.1%）	
Severe(steatosis≥60%)	0（0%）	1（2.0%）	
Cardiac-death donor livers	9(19.6%)	0(0.0%)	**<0.001**
**Operation features**			
Anhepatic period（min）	58（42-68）	52（45-59）	0.261
Operation time（h）	7.9（6.5-9.1）	6.4（5.8-7.6）	**<0.001**
RBC（u）	4.8（2.8-6.0）	5.0（2.3-8.1）	0.692
FFP（ml）	1450（1050-1800）	1000（625-1375）	**<0.001**
Orthotopic LT（n,%）	19（41.3%）	29（59.2%）	0.082
Piggyback LT（n,%）	27（58.7%）	20（40.8%）	

CLT, Conventional Liver Transplantation; IFLT, Ischemia-Free Liver Transplantation; ECD，Extended Criteria Donor; BMI, Body Mass Index; MELD, Model for End-Stage Liver Disease; RRT, Renal Replacement Therapy; MDRD4-EGFR, Estimated Glomerular Filtration Rate calculated using the equation of the Modification of Diet in Renal Disease Study Group; HCC, Hepatocellular Carcinoma; AST, Aspartate aminotransferase; RBC, Red Blood Cells; FFP, Fresh Frozen Plasma; LT, Liver Transplantation.

Table S5. Comparison of postoperative renal function of patients between ECD-IFLT Group and ECD-CLT Group after matching.

	ECD-CLT (n=46)	ECD-IFLT (n=49)	***P***
**Acute Kidney Injury**	25（54.3%）	28（57.1%）	0.784
Stage-1 AKI	11（23.9%）	22（44.9%）	**0.032**
Stage-2 AKI	2（4.3%）	4（8.2%）	0.678
Stage-3 AKI	12（26.1%）	2（4.1%）	**0.002**
**Acute Kidney Injury Severity**			
Mild AKI[a]	11（23.9%）	22（44.9%）	**0.032**
Severe AKI[b]	14（30.4%）	6（12.3%）	**0.030**
**Acute Kidney Injury Duration**			
Transient AKI[c]	11（23.9%）	24（49.0%）	**0.011**
Persistent AKI[d]	14（30.4%）	4（8.2%）	**0.006**
**Postoperative Renal Replacement Therapy**	7（15.2%）	3（6.1%）	0.190
Initial RRT[e]	4（8.7%）	2（4.1%）	0.426
Persistent RRT[f]	3(6.5%)	1(2.0%)	0.351
**Preoperative Renal Replacement Therapy**	3（6.5%）	3（6.1%）	0.631
RRT cessation[g]	0（0%）	2（4.1%）	0.495
Percentage of RRT cessation within POD 7（%）[g]	0/3（0%）	2/3（66.7%）	1.000
**Creatinine level**			
Peak creatinine within POD 7（umol/L）	92（74-218）	94（81-127）	0.791
Peak creatinine within POD 7/Baseline creatinine	1.4（1.1-2.1）	1.3（1.1-1.7）	0.507
Change in serum creatinine（umol/L）[h]	20（5-85）	28（4-43）	0.760

CLT, Conventional Liver Transplantation; IFLT, Ischemia-Free Liver Transplantation; AKI, Acute Kidney Injury; SAKI, Severe Acute Kidney Injury; RRT, Renal Replacement Therapy.

[a] Mild AKI：refers to Stage-1 AKI.

[b] SAKI: including Stage 2 AKI and Stage 3 AKI.

[c] Transient AKI: AKI lasting < 48 hours.

[d] Persistent AKI: AKI lasting ≥ 48 hours.

[e] Initial RRT: refers to patients who did not receive RRT before LT and received RRT for the first time within 7 days postoperatively.

[f] Persistent RRT: refers to the condition where RRT is required both preoperatively and postoperatively.

[g] RRT cessation：refers to patients who required RRT before LT but no longer needed RRT within 7 days postoperatively. Percentage of RRT cessation within 7 days postoperatively = Number of RRT cessation / Number of patients who received RRT treatment before surgery.

[h] Change in serum creatinine = Peak creatinine within 7 days postoperatively - Baseline creatinine before surgery.
